# COMPARING PEOPLE WITH PERSISTING POST-CONCUSSION SYMPTOMS FROM AN EMERGENCY DEPARTMENT-BASED RESEARCH SAMPLE WITH PATIENTS IN A CLINICAL REHABILITATION SAMPLE: AN EXPLORATIVE, RETROSPECTIVE ANALYSIS

**DOI:** 10.2340/jrm.v58.45004

**Published:** 2026-03-23

**Authors:** Maria I. SANDBOE, Linda FORDAL, Alexander OLSEN, Grant L. IVERSON, Helge SKIRBEKK, Mille Møller THASTUM, Jørgen Fældbæk NIELSEN, Simen Berg SAKSVIK, Toril SKANDSEN

**Affiliations:** 1 Department of Neuromedicine and Movement Science, Norwegian University of Science and Technology (NTNU), Trondheim; 2 Clinic of Rehabilitation, St. Olavs Hospital, Trondheim University Hospital, Trondheimy; 3 Department of Psychology, Norwegian University of Science and Technology (NTNU), Trondheim; 4 NorHead – Norwegian Centre for Headache Research, Norwegian University of Science and Technology, Trondheim, Norway; 5 Department of Physical Medicine and Rehabilitation, Harvard Medical School, Boston, MA; 6 Department of Physical Medicine and Rehabilitation, Spaulding Rehabilitation Hospital and the Schoen Adams Research Institute at Spaulding Rehabilitation, Charlestown, MA; 7 Mass General for Children Sports Concussion Program, Waltham, MA; 8 Home Base, A Red Sox Foundation and Massachusetts General Hospital Program, Charlestown, MA, USA; 9 Department of Nursing and Health Promotion, Oslo Metropolitan University, Oslo; 10 Norwegian Concussion Association, Oslo, Norway; 11 Hammel Neurorehabilitation Centre and University Research Clinic, Aarhus University, Aarhus, Denmark; 12 Department of Research and Development, St. Olavs Hospital, Trondheim University Hospital, Trondheim, Norway

**Keywords:** minimal head injury, mild head injury, mild traumatic brain injury, concussion, post commotio syndrome, rehabilitation, return to work, Rivermead Post-Concussion Symptoms Questionnaire, treatment-seeking patients

## Abstract

**Objective:**

To compare characteristics of people with persisting post-concussion symptoms in a sample referred for treatment and in a sample from a prospective research study.

**Design:**

Observational study.

**Subjects:**

Participants, aged 18–60 years, with persisting post-concussion symptoms 3–18 months after mild head injury, 92 with mild traumatic brain injury followed from the emergency department (“prospective emergency department sample”) and 106 patients with either mild traumatic brain injury or minimal head injury, referred to an outpatient rehabilitation clinic (“clinical rehabilitation sample”).

**Methods:**

Persisting post-concussion symptoms were defined as having British Columbia Post-Concussion Symptom Inventory scores indicating at least moderate persisting post-concussion symptoms and/or Rivermead Post Concussion Symptoms Questionnaire (RPQ) scores ≥ 12. Symptoms, functional outcome, work/school participation, resilience, and fatigue were examined at 3–18 months post injury.

**Results:**

Compared with the prospective emergency department sample, the clinical rehabilitation sample had higher education, higher RPQ scores (30 vs 17), lower Glasgow Outcome Scale Extended scores (median 6 vs 7), and more had not returned to work or school (43% vs 18%).

**Conclusion:**

Treatment-seeking patients with persisting post-concussion symptoms differed in clinically important ways from people who developed persisting post-concussion symptoms in an emergency department-based mild traumatic brain injury study. Results from studies of mild traumatic brain injury seen in the emergency department may not generalize to patients seeking specialized treatment for persisting post-concussion symptoms.

## LAY ABSTRACT

Having persisting symptoms after mild head trauma, referred to as persisting post-concussion symptoms, is not rare, yet the condition remains poorly understood. To provide better treatment, we need to accumulate knowledge on the individuals in need of healthcare for persisting post-concussion symptoms. Findings from studies of patients seen in emergency departments after an acute mild head injury may not be representative for patients seen in other settings, such as rehabilitation clinics. This study compared 2 different groups of individuals with persisting post-concussion symptoms, 1 comprising patients referred for treatment at a specialized rehabilitation clinic and 1 comprising individuals followed from an Emergency Department. We found that the patients referred to the rehabilitation clinic had more severe symptoms and more difficulties in daily life after injury, despite having sustained a milder injury. These findings highlight the need for more research on those seeking specialized healthcare to improve clinical care and potentially prevent prolonged symptoms and problems.

Most individuals with mild traumatic brain injury (mTBI) recover without lasting complications, but a significant minority develop persisting symptoms, commonly described as persisting post-concussion symptoms (PPCS), which may endure for months or even years following the initial injury (1). PPCS includes physical, cognitive, and emotional symptoms that can lead to prolonged work absenteeism (2) and diminished social participation and quality of life (3). For those seen in the emergency department (ED) and followed over time, around 10–30% report PPCS 3–6 months post injury (1), and in some studies the percentage is higher in part due to the operational definition of PPCS and the research setting (4, 5). Consequently, there is a pressing need to develop evidence-based strategies for the prevention and management of PPCS, and substantial efforts have been directed toward developing prediction models based on admission characteristics to identify individuals at risk (6).

Important longitudinal studies recruiting patients who have been evaluated in emergency departments in the United States, such as Transforming Research and Clinical Knowledge in TBI (TRACK-TBI), and in Europe, such as CENTER-TBI (Collaborative European Neuro Trauma Effectiveness Research in TBI), are yielding valuable findings relating to outcome from mTBI (4, 7). It is, however, not well understood whether patients who present to the emergency department after a mild head injury and are followed in prospective research studies are representative of those who seek specialized healthcare for PPCS. A substantial percentage of people with mTBI do not seek medical attention immediately after the injury, if they seek care at all (8), and many only see their general practitioner (GP) (9, 10). Moreover, the presence and severity of PPCS are not consistently associated with the severity of the injury to the head and brain (11), and, notably, PPCS is also reported after head injuries not clearly meeting the criteria for mTBI (e.g., with no clear alteration of mental state) (10, 12), and these patients are commonly excluded from studies of mTBI and are thus under-represented in research. From a clinical perspective, physicians and therapists are frequently confronted with considerable heterogeneity and multifaceted clinical presentations in individuals seeking care for PPCS. A particularly perplexing aspect is when patients often report a substantial symptom burden, which seems to stand in contrast to the low-impact nature of the head trauma experienced at the time of injury.

This study builds upon our previous research on characteristics of clinical patients with PPCS after minimal and mild TBI (10), and the epidemiology and outcome of mTBI in a prospective study (13), utilizing partly overlapping data. We compared patients with PPCS from 2 different samples within the same geographical area, as follows: (*i*) patients who were referred to a specialized outpatient clinic after mTBI or mild head injury, and (*ii*) people with mTBI who were followed from EDs as part of a prospective study. We aimed to assess whether characteristics differed between patients referred for treatment of PPCS and individuals who developed PPCS during the course of participating in a prospective cohort study.

## METHODS

### Settings and participants

Study subjects came from 2 distinct samples, and were either: (*i*) patients referred for specialized evaluation and treatment at an outpatient rehabilitation clinic and participants in the Trondheim PPCS study, or (*ii*) participants in a prospective ED-based study, the Trondheim mTBI follow-up study (13). Based on common inclusion criteria, eligible subjects from these 2 samples were included in the present study, and the data were merged into a common dataset. The 2 studies recruited patients from the same geographical area. Outcome definitions and study criteria for the 2 samples are described below and summarized in Table I.

**Table I:** Outcome definitions and study criteria

Variables	Prospective ED sample	Clinical rehabilitation sample
Inclusion criteria for diagnosis of PPCS for the present study	PPCS at 12 months, or 3 months if missing at 12 month	PPCS at baseline interview 3–18 months post-injury
Head injury criteria	mTBI according to WHO criteria	mTBI according to WHO criteria or minimal head injury according to HISS
PPCS criteria	3 or more moderate to severe post-concussion symptoms on the BC-PSI, a BC-PSI total score of ≥13, and/or an RPQ total score ≥ 12	At least 1 post-concussion symptom of at least moderate severity and 3 or more mild symptoms on the RPQ, or/and an RPQ total score ≥ 12 at baseline
Post-concussion symptoms measure	RPQ BC-PSI	RPQ
Functional outcome	GOSE	GOSE

ED: emergency department; PPCS: persistent post-concussion symptoms; mTBI: mild traumatic brain injury; WHO: World Health Organization; RPQ: Rivermead Post Concussion Symptoms Questionnaire; BC-PSI: British Columbia Post-Concussion Symptom Inventory; GOSE: Glasgow Outcome Scale Extended.

### The Clinical Rehabilitation Sample, derived from the Trondheim PPCS study

The Trondheim PPCS study is a prospective observational study comprising all consenting patients treated for PPCS and post-traumatic headache at the outpatient hospital-based service, Clinic of Rehabilitation, St. Olav’s University Hospital (publicly funded). Patients are referred to this service by their general practitioner (GP) or hospital physicians for specialized evaluation and treatment. The median time from referral to the first appointment is 6 weeks; some are seen within 3 weeks of referral. The GPs have access to an electronic clinical handbook that includes some recommendations regarding mild head injuries, but specific Norwegian guidelines for PPCS are lacking. Patients are eligible for inclusion if they have sustained a mild head injury, either mTBI using the World Health Organization (WHO) Collaborating Centre Task Force on Mild Traumatic Brain Injury criteria for mTBI described below, or minimal head injury (MHI) using the Head Injury Severity Scale (HISS) which is: Glasgow Coma Scale (GCS) score 15, no loss of consciousness (LOC), no post-traumatic amnesia (PTA), and no intracranial findings on imaging (14). Further, the participants (*i*) experienced post-concussion symptoms within the first week after injury, and (*ii*) they reported at least 1 moderate or severe symptom, or they reported 3 or more mild symptoms on the Rivermead Post Concussion Symptoms Questionnaire (RPQ), at the first evaluation at the clinic. Patients were not included if they had problems communicating in Norwegian or English, if the physician judged their symptoms to be caused by a co-occurring condition not related to the head injury, or if they had severe psychiatric, neurological, medical, or substance abuse disorders that would complicate evaluation of PPCS or participation in the study.

Inclusion and exclusion criteria, as well as criteria for MTBI or MHI, were evaluated by the treating physician, and patients were thereafter invited to participate. Consent was given electronically, separate from the visit. Data were collected using a semi-structured interview at the first visit at the clinic and entered into a data repository, including demographic information, injury-related information, and evaluation of current symptoms. Questionnaires measuring fatigue and resilience were sent electronically after consent. In the study period, around 90% of the patients evaluated at the clinic, and deemed eligible for the study, consented to participate in the study.

In the present study, participants included from the Trondheim PPCS study were labelled the *Clinical Rehabilitation Sample.* Participants included in the clinical rehabilitation sample were 18–60 years old and had been enrolled in the PPCS study from February 2019 to April 2023. Their RPQ total score had to be ≥ 12 at their initial clinic visit, which occurred between 3 and 18 months post-injury. For a few patients the RPQ score was missing, but they were included based on a high symptom burden documented in their medical records.

### The Prospective ED Sample, derived from the Trondheim mTBI follow-up study

In the Trondheim mTBI follow-up study, participants were enrolled from 2 different EDs as follows: (*i*) a general practitioner staffed outpatient clinic, the Trondheim Municipal Emergency Clinic; and (*ii*) a level one trauma centre at St. Olav’s Hospital, Trondheim University Hospital. Both EDs are publicly funded and together serve as the facilities in the area where patients with acute head injuries can present for care 24 h a day. In the ED, patients are provided with an information sheet listing precautions for the first few days after mTBI, as well as the expected positive outcome.

From April 2014 to December 2015, participants (aged 18–60 years) with MTBI were enrolled within 72 h after TBI, which was defined as “an alteration in brain function, or other evidence of brain pathology, caused by an external force” (15). The mechanism of injury was categorized into fall, traffic accident, violence, bicycle, hit by, or hitting, an object, and other. “Hit by, or hitting, an object” was used to describe a direct impact to the head, when the head was hit by, or struck an external object, surface, or body part. Patients were included if they met any of the following criteria: witnessed LOC or self-reported amnesia for the event or the time period after the event, showed objective neurological deficits, or had a visible traumatic brain lesion on head CT. *Mild* TBI was thereafter defined using the World Health Organization (WHO) Collaborating Centre Task Force on Mild Traumatic Brain Injury definition: (*i*) GCS score 13–15, (*ii*) LOC < 30 min, and (*iii*) PTA < 24 h (16). Participants were excluded if they were not living in Norway or not able to communicate in Norwegian, had medical or psychiatric conditions that could influence follow-up, or had other major trauma. Details have been described previously (13). Potential participants were contacted within 72 h of injury. Medical records and CT results were evaluated, and patients were further interviewed to ensure eligibility. Data were collected at baseline, and at 2-week, 3-month, and 12-month follow up. Pre-injury and injury-related data were collected from interviews and questionnaires as previously described (13). The British Columbia Post-Concussion Symptom Inventory (BC-PSI) and the Glasgow Outcome Scale-Extended (GOSE) were administered in telephone interviews at 3 and 12 months after injury. The RPQ, Fatigue Severity Scale (FSS), and the Resilience Scale for Adults (RSA) were administered as questionnaires sent by mail for most people and by telephone interview in a few cases. In a prior study, participants in the Trondheim MTBI follow-up study were shown to be representative of patients seeking acute care after mTBI (13).

In the present study, participants included from the Trondheim MTBI follow-up study were labelled the *Prospective ED Sample.* Participants were included in the prospective ED sample if they had PPCS at 3 or 12 months following injury (see details below) and were aged 18–60 years at the time of evaluation. Criteria for PPCS were BC-PSI (17) scores indicating moderate PCS (described below), a total score on the RPQ ≥ 12, or both. No firmly established recommendation exists for a specific RPQ cutoff score, but other studies have used values in a comparable range (5). RPQ and BC-PSI data from the 12-month follow-up were used in the present study. If the 12-month follow-up was missing, data from the 3-month follow-up were used.

### Measures

Post-concussion symptoms were evaluated with the BC-PSI and the RPQ. The BC-PSI is a self-report questionnaire including 13 symptoms: headache, dizziness/light-headedness, nausea, fatigue, noise sensitivity, irritability, sadness, nervousness, temper problems, concentration ability, memory problems, reading difficulties, and sleep difficulties. The respondent rates the 13 symptoms on a scale from 0–5 that measures both the frequency and intensity of the symptom for the past 2 weeks. Multiplication of the frequency and intensity ratings creates a single product-based score for each symptom, and these product-based scores are then converted to item scores of 0–4 (0–1 = 0, 2–3 = 1, 4–6 = 2, 8–12 = 3, and 15+ = 4), where a higher item score is indicative of greater frequency and severity of the symptom (17). PPCS was defined as reporting 3 or more symptoms measured as an item score of 3 or 4, or a total BC-PSI score of ≥ 13. The RPQ is a 16-item questionnaire rating the presence and intensity of 16 symptoms during the last 24 h, compared with before injury (18), including headaches, dizziness, nausea/vomiting, noise sensitivity, sleep disturbance, fatigue, irritability, feeling depressed/tearful, feeling frustrated/impatient, forgetfulness, poor concentration, taking longer to think, blurred vision, light sensitivity, double vision, and restlessness. Each symptom is rated on a scale from 0–4, where 0 equals “not a problem”, 1 equals “no more of a problem”, and 2, 3, and 4 equal mild, moderate, and severe problems. Scores > 1 are summed into a total score. The median time from referral to the first appointment is 6 weeks; some are seen within 3 weeks of referral.

Work status following injury was categorized into full-time work/school or not. The work status variable is hereafter presented as full-time work or school for short. Full-time work was defined as ≥ 37.5 h per week, and full-time school was defined as ≥ 25 h per week. The subcategory “other” included pensioners, people on parental leave, and voluntary work. Psychiatric symptoms and problems, such as anxiety or depression, were subjectively reported and not necessarily formally diagnosed by a professional. Previous head injuries were self-reported in both studies, comprising injuries that met the WHO Task Force criteria for mTBI.

The structured interview for the GOSE was used to assess functional outcome (19). The scores 5 (lower moderate disability) and 6 (upper moderate disability) indicate distinct limitations in at least 1 life role. Scores of 7 (lower good recovery) and 8 (upper good recovery) represent a return to previous life roles, where 7 indicates that PPCS still influence daily life or mildly affect social participation. Number of headache days per month was recoded into < 1 day, 1–6 days, 7–14 days, and > 14 days. Each participant’s most commonly experienced headache intensity was categorized as mild (does not affect daily activities), moderate (affects daily activities), or severe (prevents all daily activities).

Resilience, defined as adapting well to sources of stress (20), was measured with the RSA and higher scores indicate greater resilience (20). Fatigue was measured with the FSS with 9 items rated from 1–7 and higher scores indicating greater levels of fatigue (21). The items grade the impact of fatigue on motivation, physical activity, everyday tasks, work, and social life. Clinically significant fatigue was defined as having a mean item-score of 5 or greater (22).

### Statistical analyses

Independent *t*-tests were used to compare continuous variables with normally distributed data (e.g., age) and Mann–Whitney *U* tests for continuous variables with non-normal distributions or ordinal data. Kolmogorov–Smirnov tests and Q–Q plots were used to test normality. χ^2^ tests were used for categorical variables. Evaluation of differences between groups in distributions of categorical data was based on inspection of the data. Linear regression with adjustment for age was used for the comparison of education between the 2 samples.

Some participants did not return the questionnaires, which led to missing data (e.g., the RPQ, RSA, and number of headache days in the prospective ED sample, and the RSA in the clinical rehabilitation sample). Statistical analyses were conducted to compare total symptom scores between subjects with and without missing questionnaire data. Specifically, Wilcoxon rank-sum tests were used to compare BC-PSI scores between participants in the prospective ED sample with and without RPQ scores and between those with and without data on the RSA and reported headache days. Independent Mann–Whitney *U* tests were used to compare RPQ scores between participants from the clinical rehabilitation sample with and without data on the RSA.

Effect sizes were calculated using the Cliff’s delta formula, δ = (2U/(n_x_n_y_))−1. Statistical analyses were performed using IBM SPSS Statistics, Version 29.0 (IIBM Corp, Armonk, NY, USA). Significance level was set to *p* < 0.01 to reduce the risk of type 1 errors associated with multiple comparisons.

## RESULTS

**Fig. 1:**
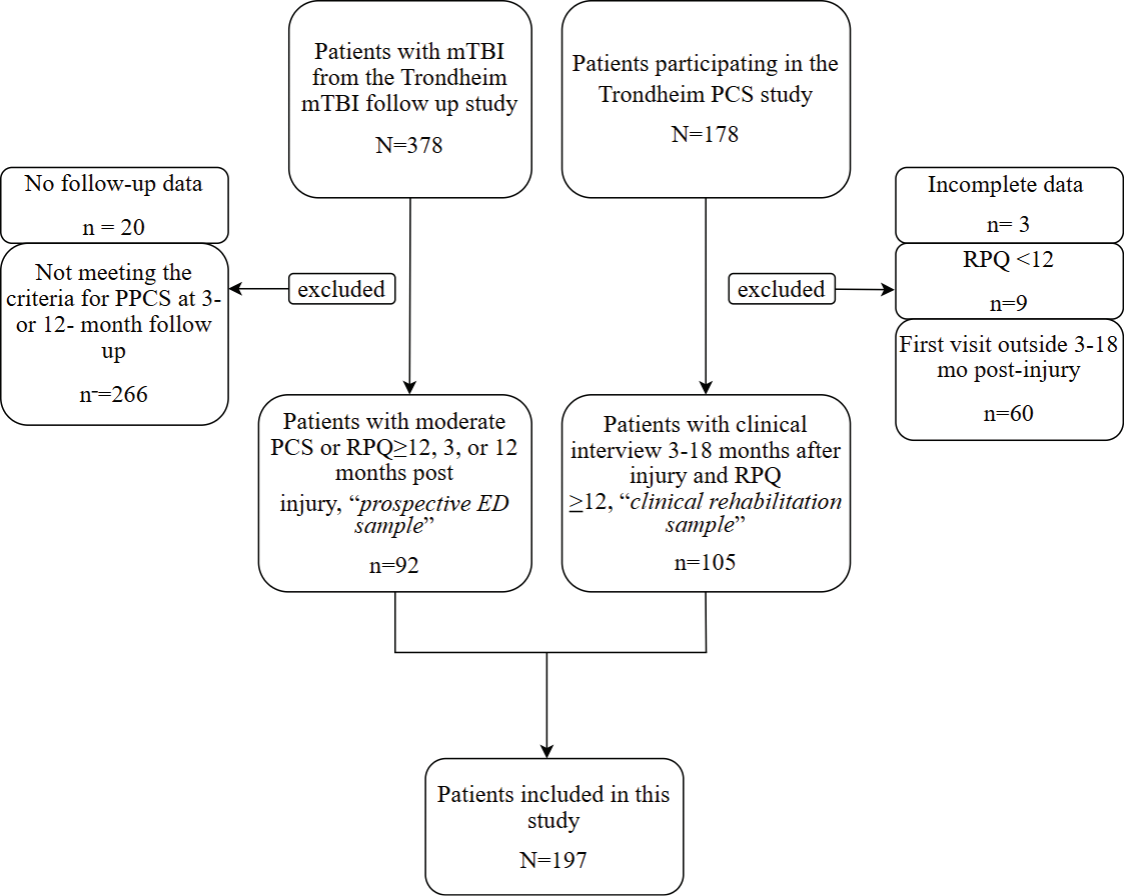
Flowchart for study inclusion. mTBI: mild traumatic brain injury; PCS: post-concussion symptoms; RPQ: Rivermead Post Concussion Symptoms Questionnaire.

There were 198 people included in this study, 106 in the clinical rehabilitation sample and 92 in the prospective ED sample (Fig. 1). A total of 72 people who participated in the Trondheim PPCS study could not be included in the clinical rehabilitation sample because outcome data were missing (*n* = 3), they were not enrolled between 3 and 18 months following injury (*n* = 60), or they did not meet the criteria for PPCS (*n* = 9). For 7 people the RPQ total score was missing due to an error in the data collection, but medical records documented a symptom burden clearly above 12 and they were thus included.

The median time from injury to data collection was 7 months in the clinical rehabilitation sample and 12 months in the prospective ED sample, where 37% were assessed at 3 months and 63% at 12 months. Fig. S1 shows the distribution of time since injury in the study samples.

### Group differences in clinical and demographic characteristics

The clinical rehabilitation sample had a significantly greater symptom burden, worse functional outcome as assessed by the GOSE, more headache days, and there was also some evidence that they more often had fatigue severity above the clinical cut-off (Table II). Between-group differences in RPQ scores and GOSE scores revealed medium effect sizes of 0.54 and 0.59. The higher symptom burden in the clinical rehabilitation sample was not related to time since injury (see Figs S1 and S2).

**Table II:** Post-injury symptoms, employment, and other outcome variables

Variables	Prospective ED sample (*n* = 92)		Clinical rehabilitation sample (*n* = 105)		*p*-value
	*n*	Value	*n*	Value	
Total RPQ score, median (IQR)	52	17 (11–24)	95	30 (24–37)	< 0.001
Total emotional items RPQ, median (IQR)	62	5.5 (2–9.25)	63	8 (6–12)	<0.001
BC-PSI total score, median	79	18			
GOSE score, median (IQR)	90	7 (6–7)	88	6 (5–6)	< 0.001
Number of headache days	55		85		< 0.001
No headache, *n* (%)		8 (15)		7 (8)	
<1 day, *n* (%)		5 (9)		0	
1–6 days, *n* (%)		19 (35)		11 (13)	
7–14 days, *n* (%)		12 (22)		3 (4)	
More than 14 days, *n* (%)		11 (20)		64 (75)	
Employment, *n* (%)	91		86		<0.001
Full-time work, *n* (%)		41 (46)		8 (9)	
Part-time work, *n* (%)		9 (10)		25 (29)	
Full-time school, *n* (%)		16 (18)		12 (14)	
Part-time school, *n* (%)		2 (2)		4 (5)	
No work/school, *n* (%)		16 (18)		37 (43)	
Other, *n* (%)		7		0	
Fatigue, *n* yes (%)	57	19 (33)	70	36 (51)	0.041
Fatigue Severity Scale, mean total score (SD)	57	39 (13)	70	44 (12)	0.035
Resilience Scale for Adults, median total score (IQR)	58	170 (151–188)	57	158 (141–176)	0.028

ED: emergency department; IQR: interquartile range; mTBI: mild traumatic brain injury; RPQ: Rivermead Post Concussion Symptoms Questionnaire; BC-PSI: British Columbia Post-Concussion Symptom Inventory; GOSE: Glasgow Outcome Scale Extended; SD: standard deviation. The RPQ scores sum the symptom severity ratings for the scale and for the emotional items.

There was a marked difference in the proportion of individuals who returned to work or school. In the clinical rehabilitation sample, only 23% participated in full-time work or school at their first presentation to the clinic (3–18 months after injury). In contrast, in the prospective ED sample, a quarter had returned to full-time work or school at the 3-month (23%) and the majority by the 12-month (62%) follow-up. Resilience Scale scores were lower in the clinical rehabilitation sample compared with the prospective ED sample (*p* = 0.028), although not significant at the *p* < 0.01 level (see Table II).

The 2 samples differed in terms of some pre-injury characteristics. Education was significantly higher in the clinical rehabilitation sample compared with the prospective ED sample, and there were more women (*p* = 0.017), although not this finding was not significant at the *p* < 0.01 level (Table III). Those in the clinical rehabilitation sample were more likely to have a pre-injury history of mTBI. Pre-injury employment, including being a student, was high in both groups. Pre-injury psychiatric problems and headaches were commonly reported in both groups.

**Table III:** Pre-injury characteristics of the study samples

Variables	Prospective ED sample (*n = 92*)		Clinical Rehabilitation sample (*n = 105*)		*p*-value
	*n*	Value	*n*	Value	
Age, mean (SD)	92	33 (14)	106	37 (13)	0.042
Sex, *n* women (%)	92	50 (54)	106	75 (71)	0.017
Full-time work or school, *n* (%)	91	70 (87)	105	95 (91)	0.418
Reduced work hours, *n* (%)		7 (8)		4 (4)	
Education, median total years (IQR)*	92	13 (12–16)	90	16 (14–17)	0.003**
Psychiatric problems, *n* yes (%)***	92	30 (33)	94	35 (37)	0.508
Headache, *n* yes (%)	92	40 (44)	105	47 (45)	0.856
Previous mTBI, *n* yes (%)	92	26 (28)	97	46 (44)	0.003

* Basic education in Norway is 13 years.

** Adjusted for age with linear regression.

*** Subjectively reported, such as anxiety and depression.

ED: emergency department; SD: standard deviation; IQR: interquartile range; mTBI: mild traumatic brain injury.

Regarding injury-related characteristics, all participants in the prospective ED sample met criteria for mTBI, in accordance with the study criteria, whereas in the clinical rehabilitation sample 57% met criteria for mTBI and 43% for MHI (Table IV). Falls were the most common mechanism of injury in both study samples. Being hit by an object was common in the clinical rehabilitation sample but not in the prospective ED sample. In the prospective ED sample, head CT was performed more often, and there was a greater proportion of positive CT findings. Being evaluated only by the GP following injury was common in the clinical rehabilitation sample.

**Table IV:** Injury-related characteristics

Variables	Prospective ED sample (*n* = 92)		Clinical rehabilitation sample (*n* = 105)		*p*-value
	*n*	Value	*n*	Value	
Time since injury, median (IQR) (months)	92	12 (3–12)	105	7 (4–9)	0.106
3 months, *n* (%)		34 (37)			
12 months, *n* (%)		58 (63)			
Injury severity	92		91		
mTBI, *n* (%)	92	92 (100)	52	52 (57)	
Minimal head injury, *n* (%)		0	39	39 (43)	
GCS score	88				
15		69			
14		18			
13		1			
Mechanisms of injury	92		105		<0.001
Fall, *n* (%)		41 (45)		43 (41)	
Traffic accident, *n* (%)		13 (14)		13 (12)	
Violence, *n* (%)		19 (21)		9 (9)	
Bicycle, *n* (%)		12 (13)		6 (6)	
Hit by, or hitting, an object, *n* (%)		3 (3)		33 (31)	
Other, *n* (%)		2		1	
CT findings	79		62		0.158
Normal, *n* (%)		62 (67)		53 (53)	
Facial or cranial fracture, *n* (%)		6 (7)		2 (2)	
Intracranial finding, *n* (%)		5 (5)		4 (4)	
Fracture and intracranial finding, *n* (%)		6 (7)		0	
No CT, *n* (%)	92	13 (14)	105	43 (41)	< 0.001
PTA, *n* yes (%)	92	100	105	50 (47)*	< 0.001
Level of care	92		98		< 0.001
GP, *n* (%)		0		40 (41)	
ED with no admission, *n* (%)		55 (60)		42 (44)	
Observation < 24 h, *n* (%)		15 (16)		3 (3)	
Admitted > 24 h, *n* (%)		22 (24)		12 (12)	

* Difficult to assess in 8.5% of cases. ED: emergency department; IQR: interquartile range; mTBI: mild traumatic brain injury; GCS: Glasgow Coma Scale, CT: computed tomography; PTA: post-traumatic amnesia; GP: general practitioner.

To assess the potential impact of missing questionnaire data, we examined whether those who did not return questionnaires differed in PPCS severity from those with complete responses. First, in the clinical rehabilitation sample, the RSA score was missing in 46% of cases, and there was no significant difference in RPQ scores between patients with and without data on the RSA (mean RPQ score 33 vs 29, *p* = 0.226). Second, in the prospective ED sample, 43% of participants had not completed the questionnaires including the RPQ, RSA, and number of headache days and there was no significant difference in the median BC-PSI scores between those with and without questionnaire data (all 19 vs 18, *p* = 0.667).

We also explored whether the inclusion of people with MHI in the clinical rehabilitation sample influenced the results because, according to the inclusion criteria, there were no individuals with MHI in the prospective ED sample. We therefore conducted subgroup analyses including only people with mTBI as defined by WHO criteria (*n* = 52 [57%]) in the clinical rehabilitation sample in the group comparisons. These analyses revealed similar results (Table SI).

## DISCUSSION

This study compared individuals with PPCS from a clinically referred outpatient sample and a prospective ED-based research sample, revealing differences in pre-injury and injury-related characteristics, as well as in the initial level of acute healthcare received. Even though the prospective ED sample had signs of more severe injury, the clinical condition of PPCS was more severe in the clinical rehabilitation sample, characterized by higher symptom burden, lower functioning, and less work/school participation.

### Symptom burden

The total RPQ scores were considerably higher in the clinical rehabilitation sample than in the prospective ED sample (see Table II), and higher than reported in many prospective studies of patients followed from the ED (23). However, symptom burden in 2 Danish studies of PPCS patients, to a large degree recruited from a clinical setting, was more similar to our findings (24–26), indicating that symptom severity may depend on the recruitment setting.

As expected, there was a high number of monthly headache days in the clinical rehabilitation sample, reflecting the high symptom burden. Headache is one of the most frequently reported symptoms among patients with PPCS, and it is considered to be the most disabling post-concussive symptom (27). Moreover, fatigue was frequently reported in both samples and was more common and more severe in the clinical rehabilitation sample, corresponding to the high symptom burden. The median time from referral to the first appointment is 6 weeks; some are seen within 3 weeks of referral. The greater severity of PPCS and post-traumatic headache observed in the clinical rehabilitation sample was anticipated and is likely due to the substantial functional impact of symptoms, prompting referral to specialized services by GPs. Consequently, compared with the prospective ED sample, the clinical rehabilitation sample reflects a selectively referred population. Importantly, this group may more accurately represent patients with PPCS who require rehabilitation. These findings highlight that the construct of “PPCS” varies across clinical settings and that characteristics of individuals presenting to the ED after mTBI may not generalize to those later referred to specialized rehabilitation for persistent symptoms.

Symptom burden appeared unrelated to traditional indicators of injury severity. In total, 41% of individuals in the clinical rehabilitation sample had not visited the ED, which could be indicative of a milder head injury. Moreover, in the clinical rehabilitation sample, reports of PTA were less common, as was triage to, and findings on, head CT. Many individuals from the clinical rehabilitation sample (43%) did not meet the mTBI criteria applied in the prospective ED sample, yet patients in the clinical rehabilitation sample suffered from a greater symptom burden (12, 28).

This finding contributes to the growing body of evidence suggesting that head injuries not meeting traditional mTBI criteria are nonetheless relevant to research and clinical practice relating to mild head injury. We advocate for the inclusion of such injuries in studies investigating PPCS.

### Post-injury employment and functional outcome

Importantly, participation in work or school was considerably lower in the clinical rehabilitation sample than in the prospective ED sample. The association between high symptom burden and incomplete return to work has been documented in previous studies (7, 29), and the clinical rehabilitation sample reported incomplete return to work to an even greater extent than reported in studies with participants recruited from both EDs and clinical settings (2, 29, 30). The low return to work rate in a sample referred for specialized healthcare was not surprising, given that extended periods of sick leave often lead to increased utilization of rehabilitation services, and healthcare systems are encouraged to incorporate vocational rehabilitation into clinical care. The economic, societal, and individual burden associated with PPCS underscores the critical need for accessible and effective healthcare services tailored to this patient population. Earlier and more precise identification, treatment, and follow-up could likely reduce the burden and improve quality of life for these patients (14).

In contrast to the low return to work rates found in the clinical rehabilitation sample, despite having PPCS, 63% of individuals from the prospective ED sample reported full-time return to work or school after injury. These figures are more consistent with findings from previous studies (2) and suggest that many individuals with PPCS after mTBI are able to maintain employment or academic engagement when symptoms remain within a tolerable range.

Functional outcome, as measured with the GOSE, was lower among participants in the clinical rehabilitation sample. In contrast, the GOSE scores in the prospective ED sample were consistent with previous findings from research cohorts (12, 31). Because the GOSE incorporates work and social participation, it is closely linked to post-injury employment. Therefore, the worse functional outcomes observed in the clinical rehabilitation sample are expected, given their greater symptom burden and lower rates of return to work. Accordingly, the clinical rehabilitation sample likely has worse functional outcomes than mTBI patients included in many prospective research studies. Return‑to‑work strategies may therefore be a critical component of the rehabilitation process, particularly for patients similar to those in the clinical rehabilitation sample, and may support better functional outcomes and greater participation in social roles.

Resilience appeared to be lower among individuals in the clinical rehabilitation sample than those in the prospective ED sample. An inverse relationship between resilience and PPCS has been documented by previous studies, showing lower resilience in people with PPCS than in people sustaining mTBI patients who did not develop PPCS (32–34). Notably, a previous study of a sample partly overlapping the present ED sample found that resilience was lower in patients with PPCS at 3 months, and in patients who reported PPCS at both 3 and 12 months, compared with patients who did not develop PPCS (33, 35). A lower resilience score reflects a reduced capacity to adapt to psychosocial adversities and negative life events, which may, in turn, impact participation in work or school (20). Although the finding in the present study did not reach significance at the *p* < 0.01 level, together with evidence from previous research (36) these findings support further investigation into whether treatment programmes for PPCS should incorporate strategies aimed at strengthening resilience and psychological adjustment following mTBI. Early detection of individuals with lower resilience may help identify those at heightened risk of developing disabling PPCS (32). Such information could also guide targeted interventions, because resilience‑focused approaches have demonstrated beneficial effects in prior studies (32, 36).

### Demographic and pre-injury differences

Women represented more than half of the participants in both samples, with the highest proportion in the clinical rehabilitation sample. This over-representation in the clinical rehabilitation sample aligns with previous research, indicating that female sex is a risk factor for developing PPCS (35). However, the greater number of women in the clinical rehabilitation sample may also reflect a greater tendency among women to seek healthcare (26). Moreover, and somewhat unexpectedly, the level of education was higher in the clinical rehabilitation sample. This contrasts with earlier findings that link lower education to an increased risk of PPCS (23) and delayed return to work (37). It is possible that some individuals with higher education may experience greater health-related concern (38), or hold job roles with cognitive demands that are more affected by persistent symptoms (26). Moreover, people with higher education are more likely to access specialist medical care (39). Associations between education and PPCS are likely multifactorial and complex, and require further investigation.

The higher reported frequency of previous mTBI in the clinical rehabilitation sample is noteworthy and may suggest that a past mTBI might confer increased vulnerability for worse outcomes from subsequent mild head injuries, in line with a recent study (40), although this is speculative and could not be examined in the present study. It is also possible that people with prior injuries navigate the healthcare system a little differently. They might be more likely to be referred for specialty services – at their request or because their physician is aware of the prior injury and decides a specialty referral is indicated. That said, self‑reported history of mTBI is susceptible to recall bias (41) and variation in how questions are understood (42). Therefore, this finding should be interpreted with caution.

### Strengths and limitations

A strength of this study was the opportunity to compare 2 samples of patients with PPCS recruited within the same geographical area, and the inclusion of those with MHI in addition to mTBI. There were several limitations to the present study. First, there were differences between the studies in how some data were collected. Most of the interviews in the prospective ED sample were conducted by telephone, while they were carried out in person in the clinical rehabilitation sample. Furthermore, outcome data were not collected at the same time point after injury and, potentially, participants in the prospective ED sample have had more time to recover. However, some analyses suggest that time since injury did not influence the RPQ scores in the clinical rehabilitation sample. In fact, RPQ scores appeared to be higher among individuals who experienced a longer delay between injury and their initial visit to specialized healthcare (see Fig. S2). Second, in both groups, there were missing data on some variables derived from the questionnaires that were sent after the clinical interview. However, we did not find evidence for different symptom severity in patients with missing data. Third, some participants in the prospective ED study had been lost to follow-up, and not included in the present study, hence there is a risk of attrition bias. Fourth, we do not have data on preinjury sick leave periods, which is a reported risk factor for PPCS (23). Finally, the inclusion years differed between the 2 studies. Public awareness of mild head injuries may have increased following the ED study, potentially influencing participant behaviour and reporting in the later sample.

### Conclusion

Much of the existing literature on PPCS following mTBI is based on prospective studies recruiting from EDs. We found that patients referred for PPCS to specialized healthcare at a rehabilitation clinic had more severe symptoms, worse global functional outcome, and lower return to work rates compared with those followed prospectively from the ED. In addition, the 2 groups differed in demographic characteristics and injury-related characteristics, suggesting that prospective studies recruiting patients from EDs may not be representative of people seeking specialist care for PPCS, and emphasizing the need for further research across clinical settings. Understanding the characteristics and needs of patients who seek care in outpatient rehabilitation settings, many of whom may never present to an ED, is essential for developing effective secondary prevention strategies and rehabilitation services for individuals with PPCS.

## Supplementary Material


