# Supplementary material for: COMPARING PEOPLE WITH PERSISTING POST-CONCUSSION SYMPTOMS FROM AN EMERGENCY DEPARTMENT-BASED RESEARCH SAMPLE WITH PATIENTS IN A CLINICAL REHABILITATION SAMPLE: AN EXPLORATIVE, RETROSPECTIVE ANALYSIS
Source: J Rehabil Med. 2026 Mar 23;58:45004. doi: 10.2340/jrm.v58.45004 (PMC13019437; doi:10.2340/jrm.v58.45004)
Supplement: Supplementary file 1 [file JRM-58-45004-s1.pdf]

Supplementary material has been published as submitted. It has not been copyedited, or typeset by Journal of Rehabilitation Medicine

Fig. S1. Time since injury (months).

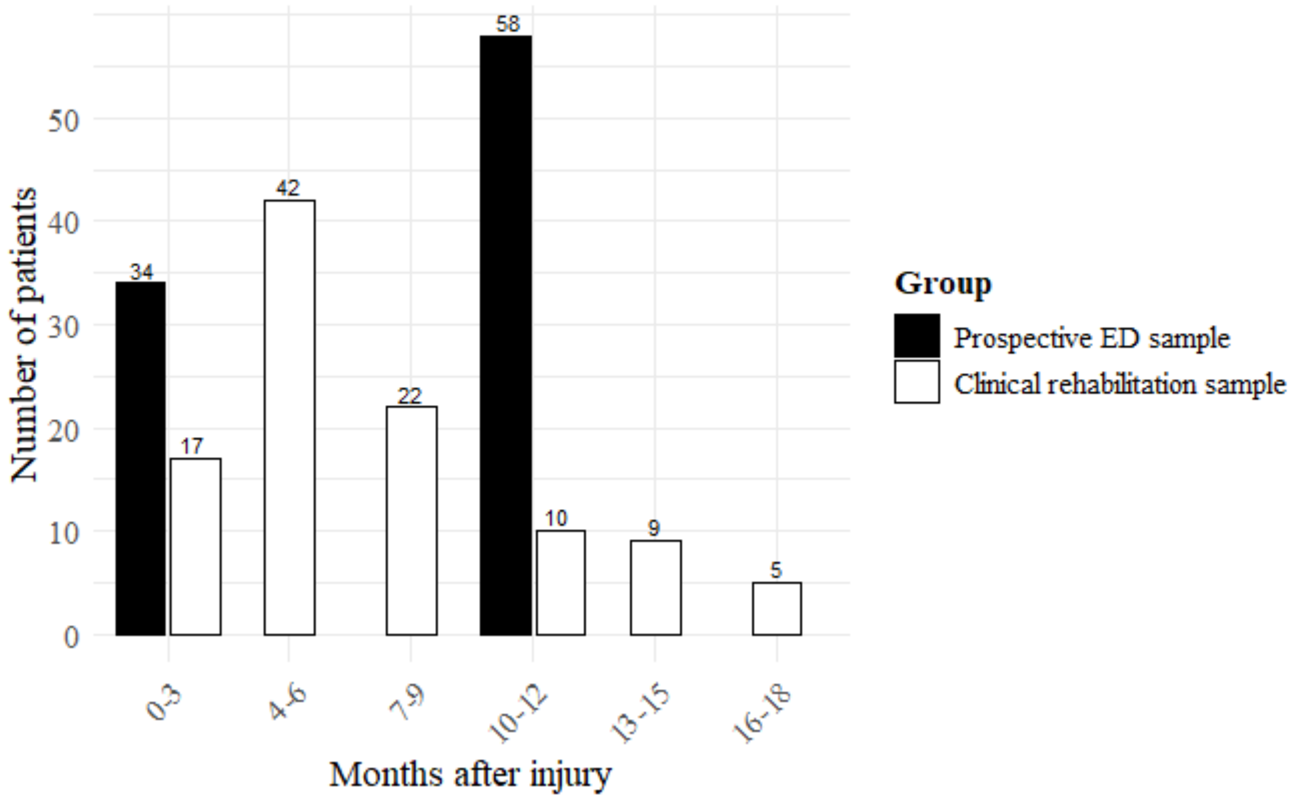

Note: ED: emergency department.

The Prospective Emergency Department sample was seen at either three months following injury (n = 34) or 12 months following injury (n = 58).

Fig. S2. Mean total symptom scores stratified by time post injury.

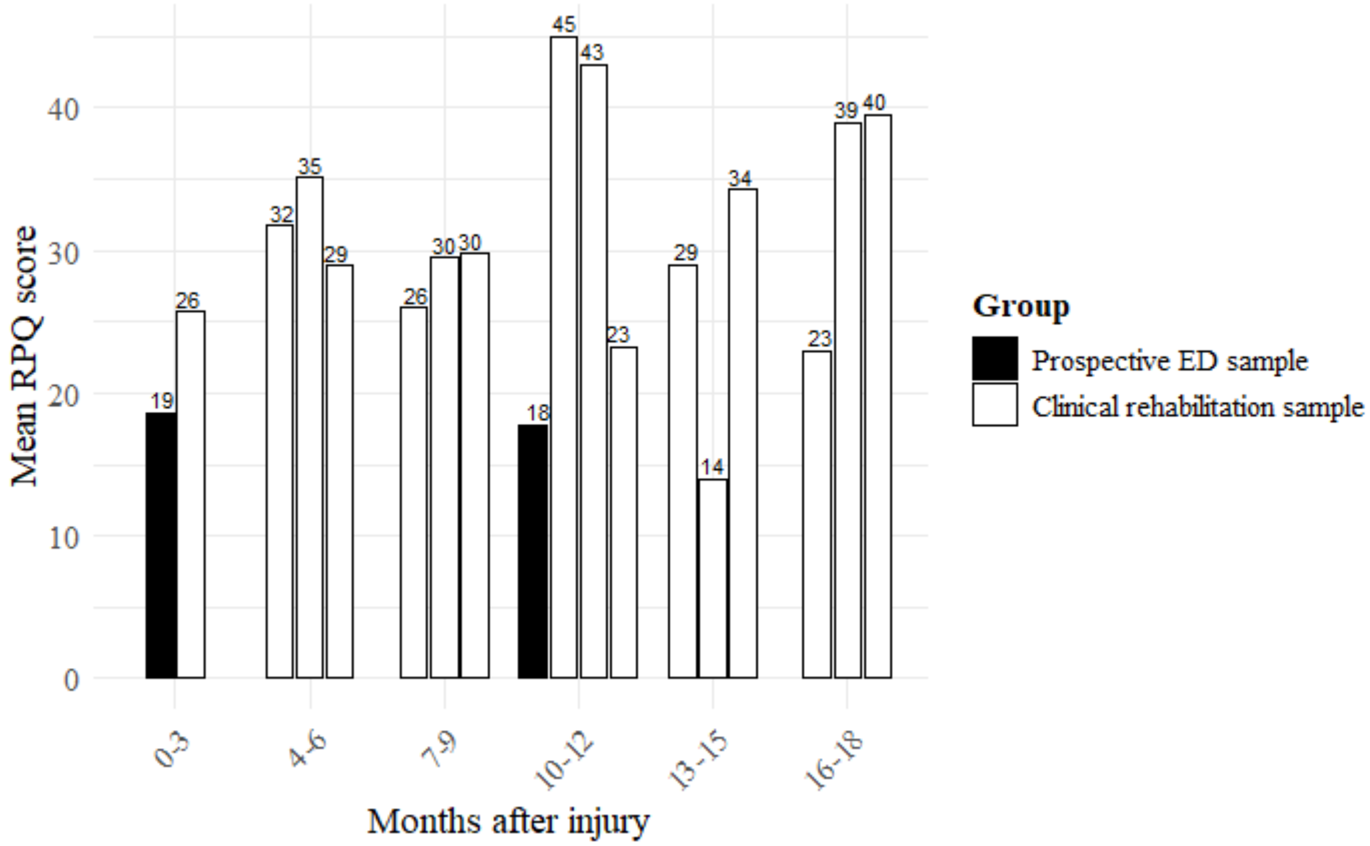

Note: ED = emergency department; RPQ = Rivermead Post Concussion Symptom Questionnaire.
